# Integration of allocentric and egocentric visual information in a convolutional/multilayer perceptron network model of goal-directed gaze shifts

**DOI:** 10.1093/texcom/tgac026

**Published:** 2022-07-08

**Authors:** Parisa Abedi Khoozani, Vishal Bharmauria, Adrian Schütz, Richard P Wildes, J Douglas Crawford

**Affiliations:** Centre for Vision Research and Vision: Science to Applications (VISTA) Program, York University, Toronto, Ontario M3J 1P3, Canada; Centre for Vision Research and Vision: Science to Applications (VISTA) Program, York University, Toronto, Ontario M3J 1P3, Canada; Department of Neurophysics Phillips-University Marburg, Marburg 35037, Germany; Centre for Vision Research and Vision: Science to Applications (VISTA) Program, York University, Toronto, Ontario M3J 1P3, Canada; Department of Electrical Engineering and Computer Science, York University, Toronto, ON M3J 1P3, Canada; Centre for Vision Research and Vision: Science to Applications (VISTA) Program, York University, Toronto, Ontario M3J 1P3, Canada; Departments of Psychology, Biology and Kinesiology & Health Sciences, York University, Toronto, Ontario M3J 1P3, Canada

**Keywords:** allocentric, convolutional network, egocentric, multilayer perceptron, saccades

## Abstract

Allocentric (landmark-centered) and egocentric (eye-centered) visual codes are fundamental for spatial cognition, navigation, and goal-directed movement. Neuroimaging and neurophysiology suggest these codes are initially segregated, but then reintegrated in frontal cortex for movement control. We created and validated a theoretical framework for this process using physiologically constrained inputs and outputs. To implement a general framework, we integrated a convolutional neural network (CNN) of the visual system with a multilayer perceptron (MLP) model of the sensorimotor transformation. The network was trained on a task where a landmark shifted relative to the saccade target. These visual parameters were input to the CNN, the CNN output and initial gaze position to the MLP, and a decoder transformed MLP output into saccade vectors. Decoded saccade output replicated idealized training sets with various allocentric weightings and actual monkey data where the landmark shift had a partial influence (*R*^2^ = 0.8). Furthermore, MLP output units accurately simulated prefrontal response field shifts recorded from monkeys during the same paradigm. In summary, our model replicated both the general properties of the visuomotor transformations for gaze and specific experimental results obtained during allocentric–egocentric integration, suggesting it can provide a general framework for understanding these and other complex visuomotor behaviors.

## Introduction

The visual system has 2 ways to code object location: relative to oneself (egocentric; Andersen and Buneo 2002; Crawford et al. 2011) or relative to surrounding objects (allocentric; Schenk 2006; Ball et al. 2009; Chen et al. 2011). This distinction has proven to be fundamental in accounts of the role of the hippocampus in spatial memory (Mishkin and Ungerleider 1982; Rolls 2020; Danjo 2020) and in 2-stream theories of vision (Schenk 2006; Thaler and Goodale 2011). This is also true for goal-directed movements. When egocentric and allocentric locations conflict, participants can be explicitly instructed to use one or the other cue, but normally (when stable) 2 sources of information are integrated to minimize internal noise and thus reduce end-point variability in the behavior (Byrne and Crawford 2010; Chen et al. 2011; Li et al. 2017). Numerous behavioral studies have suggested that this involves a process similar to Bayesian integration (Neggers et al. 2005; Byrne and Crawford 2010; Fiehler et al. 2014; Klinghammer et al. 2015, 2017; Li et al. 2017). However, the intrinsic mechanisms for representing and integrating these codes remain a puzzle.

Cue-conflict behavioral studies combined with theoretical modeling were used first to investigate the computational rules for allocentric and egocentric integration for reach control (Byrne and Crawford 2010; Fiehler et al. 2014; Klinghammer et al. 2015, 2017). For example, when a landmark was shifted relative to the remembered location of a reach target, reach end-points shift partially in the same direction, consistent with Bayesian integration and the outputs of a maximum likelihood estimator (Byrne and Crawford 2010). The amount of the shift was also affected by the number of relevant objects in the scene as well as scene consistency (Klinghammer et al. 2015, 2017). Neuropsychology results imply that egocentric and allocentric visual representations are segregated in the dorsal and ventral streams of vision respectively (Schenk 2006; Thaler and Goodale 2011), suggesting a need to reintegrate this information at some point in the brain. Subsequent neuroimaging studies confirmed such segregation (Chen et al. 2014) and suggested that the recombination occurs in parietofrontal cortex (Chen et al. 2018), but neuroimaging data are too coarse-grained to identify specific computational mechanisms at the cell/circuit level.

These observations have also been extended to the monkey gaze control system. As in reaching, when monkeys were trained to saccade to remembered targets in the presence of a landmark shift, their gaze endpoints shifted partially in the same direction, again implying weighted integration between egocentric and allocentric inputs (Li et al. 2017). Landmark influence was larger when it was closer to initial gaze fixation and when it was shifted away from the target. The development of this animal model then allowed for neural recordings associated with allocentric–egocentric integration for goal-directed movements. In particular, Bharmauria et al. (2020, 2021) recorded from the frontal eye fields (FEF) and supplementary eye fields (SEF) while rhesus monkeys performed the cue-conflict gaze task described above. When the landmark shifted, its influence was first observed multiplexed in FEF/SEF delay responses, and then fully integrated in the gaze motor response (Bharmauria et al. 2020, 2021).

In summary, it appears that egocentric and allocentric codes are at least partially segregated within the visual system, but then reintegrated for action in parietofrontal cortex. However, the early cellular/network mechanisms for these processes remain unknown. One reason for this knowledge gap is the lack of a theoretical framework to guide physiological studies in this area. Specifically, there is a lack of models that integrate both the complexity of the visual system and the sensorimotor system in general, and allocentric–egocentric integration in particular.

One approach to building such a theoretical framework is to deploy and analyze artificial neural networks (ANNs). But here again, there is a knowledge gap because this phenomenon requires the integration of multiple features into the final motor output. Many ANN models have attempted to represent feature interactions in the visual system, but do not consider sensorimotor transformations (e.g. Geirhos et al. 2017; Rajalingham et al. 2018; Schrimpf et al. 2018; Kar et al. 2019). Other trainable ANN models have been used to simulate visuomotor transformations by constraining their inputs and outputs to resemble the known physiology of the system (Zipser and Andersen 1988; Smith and Crawford 2005; Blohm et al. 2009), but these models treated the visual world as a single “dot.” What is needed is a more general model with the capacity to represent both multiple object features and implement the sensorimotor transformation.

The purpose of this research was to develop a network model that can (i) integrate visual features and targets in order to aim gaze, and (ii) to train the model and evaluate the output of this model against existing data obtained from an allocentric–egocentric integration task. We modeled gaze saccade because of their simplicity and the availability of relevant data for training and test our model (Bharmauria et al. 2020). We used a convolutional neural network (CNN) to represent the visual system (as a model of the ventral visual pathway), connected in series with a multilayer perceptron (MLP) network to represent the visuomotor transformation. Following the precedent of previous sensorimotor models (Smith and Crawford 2005; Blohm et al. 2009), we constrained the inputs and outputs to resemble known physiology, using fully analytic solutions (Hadji and Wildes 1990). Specifically, input layers were based on known properties of the sensory cortex (Hubel and Wiesel 1968; Carandini et al. 2005; Carandini 2006; Wang et al. 2007) and outputs based on the motor response field properties of the FEF (Knight and Fuchs 2007; Knight 2012; Sajad et al. 2015; Caruso et al. 2018). For the latter, a pretrained decoder was added to the end of the MLP to transform the motor population codes into 2D saccades. We then used final gaze displacement to train network output. For this, we used both synthetic datasets and actual (behavioral) data obtained from the cue-conflict task described above (Li et al. 2017; Bharmauria et al. 2020, 2021). Finally, we compared the network properties and outputs with the reported behavioral and neural observations relevant to allocentric–egocentric combination (Li et al. 2017; Bharmauria et al. 2020, 2021).

Our results suggest that this network captures the known behavioral and neural properties of the visuomotor system in the specific tasks tested, and thus the trained MLP transformation provides a promising framework for understanding and predicting unknown properties in the visuomotor system. Furthermore, this model has potential for generalization and application for a much wider range of scenarios involving the integration of visual features for complex visuomotor tasks.

## Method

To create our model, 4 main challenges were addressed. First, training an ANN with images requires large datasets (Goodfellow et al. 2016), which is infeasible to create considering the complexity of the experiments employed in this field. Second, to yield results that are physiologically relevant, it is essential to incorporate the known physiology into the ANN. This is particularly challenging to solve for the current question because it requires modeling both the early visual system and the visuomotor transformation for goal-directed saccades, a task that (to our knowledge) has not been attempted before. Third, it is desirable for the internal operations of the model to be interpretable, which is a lack in typical learned ANNs. Finally, it was necessary to train and validate our model against real data. To do this, we used a recent experiment in our lab to train our network (Li et al. 2017; Bharmauria et al. 2020, 2021). Therefore, in the following, we first explain the task on which we based our simulation. Then, we explain the model in a more general format. Finally, we explain the extracted data we used for our simulations and the model parameters.

### Task

**Fig. 1:**
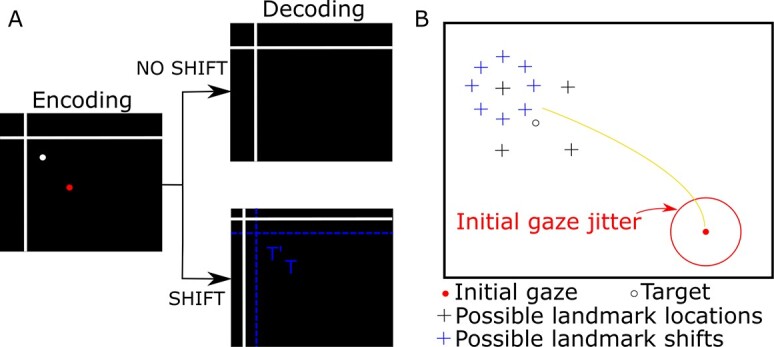
Experimental conditions. A) After initial fixation on a red dot, an image consisting of allocentric cues (2-crossing lines) and visual target (white dot) appears on the screen along with the fixation dot. We consider this image an encoding image. Monkeys have a limited time to memorize the position of the target from this image. After a random delay during which the encoding image is absent, the decoding image consisting of only visual landmarks and fixation dot appears. Based on the experiment protocol, the landmark location can be the same as the encoding image (no-shift condition) or can be different from the encoding image (shift condition). The task for the monkey is to perform a saccade toward the missing target location when the fixation signal (red dot) disappears. B) Landmark location (black crosses) was randomly selected from 4 possible positions, 11° from the target (open circle). Landmark shift was randomly selected from 8 possible shifts (blue crosses) evenly distributed on a circle 8° from the initial landmark location. Initial gaze location was jittered within a 7–12° window. Red circle indicates an example gaze jitter of 10°. Illustrated shift positions are with respect to the landmark at their center; shifts for other depicted landmarks not shown for sake of simplicity of illustration.

The details of our model were motivated based on the insights from recent experiments in the macaque gaze system (Li et al. 2017; Bharmauria et al. 2020, 2021). Therefore, it is essential to first summarize the task (Fig. 1A). Initially, a red dot appears on a screen and monkeys are trained to fixate their gaze on the red dot. After the fixation, an image, which consists of allocentric cues (two-crossing lines) and a visual target (white dot), appears on the screen (encoding image), while the fixation point remains. Monkeys have 100 ms to memorize the position of the target. After a delay, a mask appears and after a further random delay period, a decoding image appears. The decoding image consists of only the landmark. Based on the experiment protocol, the landmark location can be the same as it was in the encoding image (no-shift condition) or it can be different (shift condition). The task for the monkey is to saccade toward the missing target location when the fixation signal (red dot) disappears. Monkeys were rewarded if their gaze landed within 8°–12° of the target. The landmark locations were randomly selected from 4 possible locations distributed on the edges of a square and 11° away from the target. The shift direction was randomly selected amongst 8 uniformly distributed points on a circle 8° away from the initial landmark location. In addition, the initial gaze location (fixation) was jittered within a 7–12° window (Fig. 1B). As noted in the introduction, the results showed an influence of the landmark shift on gaze behavior as well as on SEF/FEF motor neuron population code and intrinsic coordinate frames.

### Theoretical model

#### Model overview

Our theoretical model provides a framework to study the combination of allocentric and egocentric information for goal-directed reaching movements in the brain. Toward this end, we combined 2 types of neural networks (Goodfellow et al. 2016): a CNN and a MLP to simulate different modules of this transformation in the cerebral cortex. In this section, we provide an overview of the proposed network and the input–output structure. Note that since inputs and outputs of the model represent 2-dimensional (2D) directions in eye-coordinates, we have simplified the model by implementing it in 2D.

**Fig. 2:**
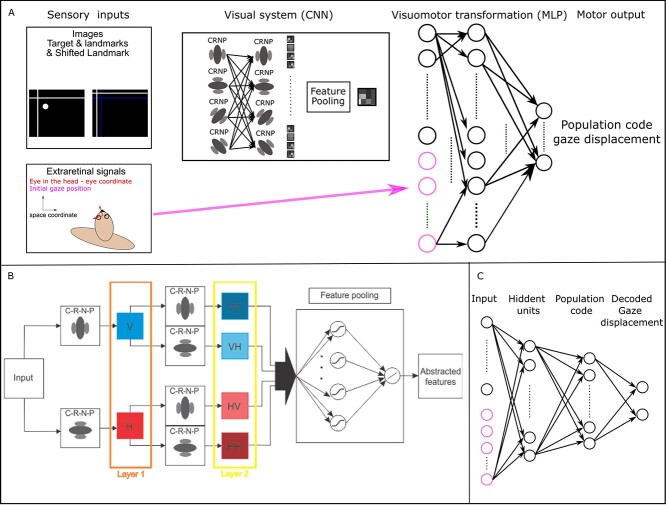
Proposed model. A) Overall network architecture. Our network consists of 4 main components. (i) Input signals: Retinal images that contain visual targets and allocentric landmarks (an example of retinal input: We simplified the retina into a 2D plane; therefore, presented images are scaled version of the presented images to the CNN (screen resolution was 1,024^*^768 and the screen was positioned 810-mm away from monkeys), which is shifted in the opposite direction of the gaze direction), and extra-retinal signals, i.e. eye position. We used 2D eye position for our simulations, (ii) a CNN to create an abstract representation of the retinal images, (iii) a MLP to implement the required sensorimotor transformations, and (iv) output signal: A decoder to transform the population code of the required movements (eye displacement) into a saccade vector. B) CNN network architecture and repeated filter mechanism*.* Layer 1 extracts local spatial features. C–R–N–P represents convolution, rectification, normalization, and spatial pooling. In addition, V and H represent vertical and horizontal filtered results, respectively. A network with only 2 layers and 2 sets of filters is depicted for illustrative purposes; 4 filters are used in actual implementation, i.e. adding 2 more orientations along the diagonals. Each feature map at layer 2 is treated as a new separate signal and is fed to the feature pooling layer. Symbol strings (e.g. VV, VH, etc.) indicate repeated filtering. The feature pooling layer consists of 2 fully connected layers: The first layer with a sigmoid transfer function and the second layer with a linear transfer function. The feature pooling layer combines the feature map from the final filtering layer to create an abstract representation of the required features. C) MLP network architecture*.* Our MLP network is a fully connected feed-forward neural network with 4 layers. The first layer is the input layer comprised of the extracted features from the images as well as extra-retinal signals. The second layer is comprised of our hidden units with sigmoid transfer functions. The third layer is the population code of the required motor movement for reaching toward the visual target. Finally, we added a read-out layer with fixed connection weights to decode the population code into 2D reach movements in Euclidean space.

Our proposed network comprises 4 main stages (Fig. 2A): inputs, a CNN, a multilayer perceptron, and output. The inputs to the network provide necessary information for the combination of allocentric and egocentric information. We feed the network with images that consist of visual targets and surrounding landmark cues, based on recent studies in the macaque, as described in the previous subsection, to provide allocentric information. These images are generated in spatial coordinates and transformed into eye-coordinates to mimic retinal projections (e.g. Klier and Crawford 1998; Klier et al. 2001). Since we did not require this model to perform 3D transformations, the only extraretinal signal that we provided was a 2D gaze signal. The second stage of our network is a CNN (Fig. 2B), which was deployed to create abstract representations of input images. The challenge here was to design the CNN as a physiologically plausible model of the early visual cortex (Hubel and Wiesel 1968; Carandini et al. 2005; Carandini 2006). In the third stage, we used an MLP (Fig. 2C) to perform the required sensorimotor transformations. The fourth stage, the output, provides the decoded saccade vectors (e.g. displacement of the eye, coded as final gaze position in eye-coordinates) from motor populations. Details of the implementation of these 4 network stages (in the format of input—output and hidden units: CNN and MLP) are provided in subsequent sections.

#### Model input–output signals

The network required 2 types of input signals: (i) retinal images (i.e. projection of the input images on the retina determined by subtracting the initial gaze locations from the images) specifying allocentric landmarks and the visual target position (which provides allocentric information); (ii) extra-retinal signals (which provide egocentric information), which in real-world conditions are required for accurate coordinate transformations (Crawford et al. 2011) and calculation of motor error (ME). The output is the signal that drives the effector (e.g. the eye in this case) from its current position to the target position (ME coded in a population format). Here, we modeled/simulated a simplified 2D representation of the retina and gaze to focus on the issue of allocentric–egocentric integration.

##### Visual inputs

The first component of the input for the network is the simulated retinal image i.e. projection of the input images on the retina determined by subtracting the initial gaze locations from the images). This component simulates a projection of the world containing both the movement target and additional landmarks on the retina. These inputs corresponded to the encoding and decoding images of the task defined in Section Task. Since we are interested in investigating the spatial component of the allocentric and egocentric combination, and save modeling of temporal aspects for future work, we process the 2 images separately as input to our network, each image having dimensions WxH, with the W and H width and height, respectively.

To simulate these inputs, we generated 2 sets of images: encoding and decoding. Each image, with the size of 200 × 200 pixels, contains two-crossing lines (a horizontal line and a vertical line, each of width 1 pixel and length extending across the entire image) as well as a 6 × 6 pixel square representing the visual target. The image intensity values for the target and lines were set to 1 and the rest of the images were set to 0. Based on the experiment protocol, there were 2 conditions for decoding images: The landmark appeared in the same location as the encoding image; or the landmark appeared in a shifted location compared with the encoding image. The specific values for the landmark and target positions for both encoding and decoding images were extracted from the actual experiment’s protocol (for further details refer to Bharmauria et al. 2020). Retinal images were calculated based on the spatial configurations of the stimuli relative to initial eye (gaze) positions. To perform this calculation, we deployed a simplified 2D equation (torsional factor is ignored for now) and calculated the retinal image by subtracting the eye position from the actual images. These retinal image values were then used as inputs the first layer of our convolutional network, described below.

##### Eye position signal

As mentioned above, extra-retinal signals are essential for performing the required reference frame transformations for various sensorimotor behaviors (Soechting and Flanders 1992; Andersen and Buneo 2002; Crawford et al. 2011). Although this transformation was not a focus here, we included a minimal extraretinal input (initial 2D gaze position) so that the model has the potential to generalize to other situations. We used the angle vector representation, $({r}_x,{r}_y)$, which is a 2D vector with components scales as the angle of rotations (Blohm et al. 2009). To encode the angular value along each axis, we used a coding mechanism analogous to activities reported in the somatosensory cortex for coding eye position (Wang et al. 2007): (i) neurons have Gaussian receptive fields with the peak indicating the preferred gaze direction; (ii) the preferred direction of neurons does not form a topographic map; and (iii) a neuron’s activity at the peak of the receptive field is monotonically increasing as the saccade amplitude grows. Similarly, we used Gaussian receptive fields randomly distributed around the orbit and gain modulated by the saccade amplitude to code the eye position in our network. This coding mechanism is formulated as:

(1) \begin{equation*} {a}_i={b}_i+{\alpha}_i\ast{r}_i, \end{equation*}

where ${a}_i$, ${b}_i$, ${\alpha}_i$, and ${r}_i$ represent the $i^{\mathrm{th}}$ unit’s activation, baseline activity, rate of the activity increase, and angular direction, respectively. Baseline activity and the rate of growth in activity are randomly picked from $(0, ma{x}_{b_i/{\alpha}_i})$. Noise is added to eye-position signal using Poisson distribution. A similar mechanism is used for coding hand or head position (King et al. 1981; Fukushima et al. 1990; Xing and Andersen 2000). Therefore, our model can be extended to include additional extra-retinal signals, if needed.

##### Motor output signal

The goal of the network is to generate movements toward a visual target. Previous work (Bruce and Goldberg, 1985; Sommer and Wurtz 2006; Sajad et al. 2015, 2016) showed that the motor neurons in FEF represent an open-end receptive field: The neural activity increases with increasing saccade amplitude. In contrast, theoretical studies suggested that cosine tuning is optimal for motor control in 3D (Kalaska et al. 1997; Kakei et al. 2001, 2003; Scott 2001). To reconcile the results of these studies with our data, we represented the response fields of our output layer as the first quarter (0–90°) of a cosine curve, according to:

(2) \begin{equation*} {a}_i={a}_0+{a}_1\times \mathit{\cos}\left({\theta}_i\right), \end{equation*}

where ${a}_0=0.5$ is the baseline activity, ${a}_1=\frac{0.5\ast \Vert \overrightarrow{M}\Vert }{M_{max}}$ is the scaling factor, angle ${\theta}_i=\Big(\frac{\overrightarrow{M}\cdot \overrightarrow{PD_i}}{\Vert \overrightarrow{M}\Vert}\Big)$ is the direction of the movement, ${M}_{max}$ is the maximum amplitude of the movement, $\overrightarrow{M}$ is the required movement, and $\overrightarrow{PD_i}$ is the preferred movement direction of the unit. We used 250 units to represent motor neurons. The maximum movement range is 150 cm. Notably, in the actual data the influence of the landmark shift was fully integrated into the FEF motor output signal, which coded a landmark-shifted gaze position in eye-centered coordinates (Bharmauria et al. 2020).

We also included an extra layer (i.e. linear decoder) to our network, called read-out, with 2 units: One coded the horizontal component of the movement, the other coded the vertical component. It has been shown that a linear decoding is suited specifically for arrays of neurons with tuning curves that resemble cosine functions (Salinas and Abbott 1994). Therefore, we determined the required weights between the population code and read-out layer by an optimal linear estimator (OLE; Salinas and Abbott 1994),

(3) \begin{equation*} {w}_{ij}={\sum}_n{Q}_{in}^{-1}\cdot{C}_{nj}, \end{equation*}

where ${w}_{ij}$ is the weight between unit $i$ at population code to unit $j$ at the read-out layer. Since we used a 2D representation, $j$ is equal to 2 and consequently ${M}_1$ represents the horizontal value of movement and ${M}_2$ represents the vertical value of movement. In addition, ${Q}_{in}$, the correlation between firing rates of neuron $i$ and *n*, and ${C}_{nj}$, the center of the mass for the tuning curve function for the neuron $n$, are calculated as

(4) \begin{equation*} {Q}_i={\sigma}_n^2{\delta}_{in}+\int d\overrightarrow{M}\cdot{a}_i\left(\overrightarrow{M}\right)\cdot{a}_n\left(\overrightarrow{M}\right), \end{equation*}

and

(5) \begin{equation*} {C}_{nj}=\int d{M}_j\cdot{M}_j\cdot{a}_n\left({M}_j\right), \end{equation*}

where ${\sigma}_n^2$ is expected neural noise. For detailed discussion and calculations, see Salinas and Abbott (1994) and Blohm et al. (2009). These weights are fixed in the network and are not changed during training. Using this strategy, we enforced a more physiologically feasible population code for our network, which enabled us to have an unambiguous interpretation of single-unit activity. In addition, using a read-out layer enabled us to have a more stable fitting procedure for training our network. More specifically, if we remove the read-out layer, we need to train the network to predict the motor population codes. The units in this layer ranged between 4 and 250. In this scenario, we needed to define a loss function that matches a vector to our desired population code. This task can become challenging based on the properties of the training data such as imbalances between different classes. To avoid dealing with such challenges, instead, we designed a fixed decoder to transform the motor population codes into the performed movement vector (in 2 dimensions). Using this method, we were able to use the least square loss function to train our network. Previous studies deployed a similar strategy (e.g. Smith and Crawford 2005; Blohm et al. 2009; Keith et al. 2010). It is noteworthy to mention that even though we simulated gaze behavior, our model is capable to be used for different body effectors (e.g. hand reaching as done in Blohm et al. 2009).

#### Hidden units

We used 2 types of networks as our hidden units. The logic is to provide a similar paradigm as the brain, with one (CNN) representing the early visual areas detecting the relevant visual features and the other (MLP) representing the higher cortical layers performing the required computations (here sensorimotor transformations) for planning the appropriate action. The parameters of our CNN were predetermined based on analytic and physiological considerations, except the feature pooling layer, which was learned. The parameters of the MLP were determined based on the training using different datasets.

#### Convolutional neural network

In recent years, CNNs have shown promising performance for a wide variety of computer vision tasks such as image classification (for a review see, e.g. Liu et al. 2020). Some studies took a step further and showed that the learned filters in convolutional networks replicate neurons’ behavior at the early visual cortex (e.g. Kriegeskorte 2015). In general, a convolutional network is comprised of several main components: convolution, rectification, normalization, and pooling layers, which typically are repeated over several layers. The first challenge in designing a CNN is to set the required components and their associated parameters properly. Here, we briefly explain the rationale behind choosing our network components and parameters based on recent physiological findings.

One of the main challenges in processing visual scenes is extracting useful information from input images (e.g. spatial and spatiotemporal patterns indicative of objects, texture, and motion). Hierarchical representations provide a powerful tool to address this challenge by progressively obtaining more abstract features at each successive layer. This incremental formation of more abstracted features yields a powerful data representation. A similar procedure is observed in the human brain. In the early visual cortex, a cascade of simple and complex cells is suggested to play a crucial role in extracting first-order stimuli in visual scenes (e.g. local measures of orientation; Hubel and Wiesel 1962; Heeger 1991).

In addition, a model with “filter-rectification-filter” has been suggested to be responsible for extracting higher-order patterns and creating a more global and abstract data representation in biological systems, e.g. texture (Baker and Mareschal 2001). Likewise, it has been shown that a similar analogy of repeated filtering and rectification can generate state-of-the-art results in computer vision applications such as dynamic texture recognition (Hadji and Wildes 1990). Here, we propose to use the same approach: repeated filtering. Figure 2B provides an overview of the convolutional network architecture. We only show a limited number of layers for illustrative purposes and only 2 filters in this figure. As mentioned above, a cascade of simple and complex cells is observed in the early visual cortex. Analogously, we employ a series of convolution, rectification, normalization, and pooling (C–R–N–P) processes to mimic the simple and complex cells’ functionality. The output of these C–R–N–P processes is then passed through the same procedures to yield the repeated filtering approach. At the last layer, we propose a cross-channel feature pooling process to create the final abstract representation of the data.

**Fig. 3:**
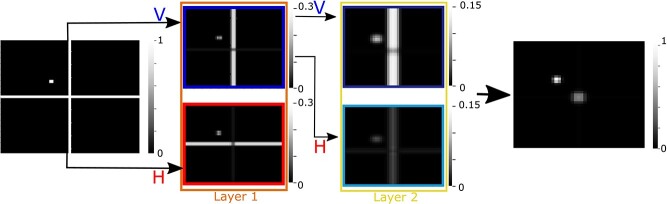
An example of processed images in a sample network with 2 layers and 2 sets of filters. V and H represent vertical and horizontal, respectively. Passing the input image through horizontal and vertical filters resulted in a separation of the horizontal and vertical lines. The target is present in both filtered images but now with different properties: More vertically oriented after passing through the vertical filter and more horizontally oriented after passing through the horizontal filter. Passing through the same filter twice (e.g. VV) resulted in almost full removal of the orthogonal line (e.g. horizontal line in VV). However, the target is still present at all the filter images with different activation level. Finally, combining all the locally extracted features by the trained feature pooling layer enabled us to detect the required features (crossing point and target position). Filter responses are represented as image brightness, with greater brightness corresponding to larger response.

Figure 3 illustrates the efficacy of the repeated filtering approach. Figures represent the local energy in images at each layer. The input is an image that contains a vertical and a horizontal line crossing and a target represented as a dot, analogous to the stimuli used in Bharmauria et al. (2020). The goal is to capture the crossing point and the target position. Here, we represent only 2 filter orientations: horizontal (H) and vertical (V). Three properties emerge after passing the image through the first layer filters: (i) the horizontal and the vertical line are separated, (ii) a gap is present at the mid-point of the line because of interference from the orthogonal lines in that vicinity, and (iii) the target appears in both filterings, because its local shape drives both. Although the target is present in both filterings, its properties are now different, e.g. the target is mainly represented by vertical components after passing through the vertical filter. In the second layer, the distinction between the 2-crossing lines becomes almost absolute. For instance, passing the input image through 2 vertically oriented filters (i.e. VV) resulted in the removal of the horizontal line. Also, the gap in the line is lessened because the interfering horizontal was suppressed from the previous layer of filtering. On the other hand, despite its different activation levels, the target is still present in all the filtered images. This result property enabled us to create an abstract representation of the target and landmark locations from input images using a feature pooling layer. In particular, our feature pooling layer detects common activated areas in all the filtered images at the second layer. Using preliminary experimentation, we found that 2 layers of repeated filtering is sufficient 4 our models. However, as shown in Fig. 3 (and explained theoretically elsewhere, Hadji and Wildes 1990), the energy in the images decreases after each layer of filtering and consequent processes, and thereby has potential to provide an automatic criterion for determining the appropriate number of layers. In the next 4 sections, we detail the operation of each of the convolutional, rectification, normalization, and pooling layers.

##### Convolution

The convolutional layer is one of the essential components of CNNs. Convolution is a linear, shift-invariant operation that performs a local weighting combination (filtering) across the input signals. The strength of such a function is that it extracts different input signal features based on the combined weights. Inspired by neuronal receptive fields in the early visual cortex, a set of oriented Gabor filters (Gabor 1946) is a popular choice (Carandini et al. 2005; Carandini 2006), which we symbolize as

(6) \begin{equation*} C\left(x;{\theta}_i,\sigma, \gamma, \lambda, \psi \right)=G\left({\theta}_i,\sigma, \gamma, \lambda, \psi \right)\ast I(x), \end{equation*}

where $I(x)$ is the input image parameterized by spatial position, *x*, ${\theta}_i$ is the orientation of the Gabor filter $G$, whereas $\sigma, \gamma, \lambda, \mathrm{and}\ \psi$ are variance, spatial aspect ratio, wavelength of the sinusoidal component, and the phase offset of the sinusoidal component of the Gabor filter, respectively. We used both cosine and sine components of the Gabor filter. In our study we only varied the orientation; therefore, for simplicity of exposition, we only explicitly notate ${\theta}_i$ for parametrizing the Gabor filter in the following.

##### Rectification

The output of the convolution contains both positive and negative values. Passing such positive and negative values through pooling can result in an attenuated response. Since we have both cosine and sine components of our Gabor filters, we deployed the energy model proposed for early visual cortex complex cells (Heeger 1991; Carandini et al. 2005) according to

(7) \begin{equation*} E\left(x;{\theta}_i\right)=\sqrt{{\left({C}_{cos}\left(x;{\theta}_i\right)\right)}^2+{\left({C}_{sin}\left(x;{\theta}_i\right)\right)}^2}, \end{equation*}

where ${C}_{cos}$and ${C}_{sin}$ are the cosine and sine components of the Gabor filtered images, respectively.

##### Normalization

We also included normalization with 2 goals in mind: First, to remove the sensitivity to local brightness; second, to prevent the rectification process from generating unbounded responses. As divisive normalization is considered a canonical process across different cortical areas (Carandini and Heeger 2012), we used a divisive form of normalization in our network according to

(8) \begin{equation*} \hat{E}\left(x;{\theta}_i\right)=\frac{E\left(x;{\theta}_i\right)}{\sum_{j=1}^nE\left(x;{\theta}_j\right)}, \end{equation*}

where $j$ ranges across the *n* filter orientations.

##### Spatial pooling

Spatial pooling is added to the network to aggregate normalized responses. This process provides a certain level of shift-invariance by abstracting the exact location of the responses in the pooling region. Similar mechanisms are observed in cortical areas (Webb et al. 2011). In our network, spatial pooling has been implemented using a low pass filter (i.e. binomial) and down-sampling with factor 2 at each layer according to

(9) \begin{equation*} S\left(x;{\theta}_i\right)={\downarrow}_{\tau}\left(B\times \hat{E}\left(x;{\theta}_i\right)\right), \end{equation*}

where $B$ is a binomial lowpass filter and ${\downarrow}_{\tau }$ is spatial down-sampling. This spatial pooling is done at the end of each layer.

##### Feature pooling

Finally, after the second layer, we included a feature pooling process. Our logic was that we created an abstraction for different features using repeated filtering (e.g. different orientations). Using the feature pooling process, we aim to create an abstraction for combined features (i.e. line-intersections). Another motivation for including feature pooling was to prevent the network from exploding, as after each filtering, the number of features will be doubled. We implemented the feature pooling process using a weighted summation of all the feature maps to yield

(10) \begin{equation*} F(x)={\sum}_{i=1}^n{w}_i\times S\left(x;{\theta}_i\right), \end{equation*}

where *n* represents the number of features (e.g. for a network with 4 orientations in each layer and 2 layers *n* will be 16). The weights ${w}_i$ are trained to detect the intersection of the 2 lines and the target. To train the weights, we created a dataset in which the inputs are the images used to train the network and the outputs are the images created by the multiplication and normalization of the final features after the second convolution (e.g. the third column in Fig. 3). Note that in our network, this feature pooling is only employed at the end of the second layer; however, for a network with more layers this mechanism may be exploited at the end of each layer (for further details see Hadji and Wildes 1990). We complete processing at this layer by transforming the representation of the images into a vector format by sequentially concatenating the image rows (flattening). This flattened feature is fed to our MLP alongside initial gaze position data. Such flattening is typical when convolutional layer results are fed to fully connected layers and does not impact the signal content, even as it makes the format more amenable to fully connected processing.

#### Fully connected layers

We used a physiologically inspired fully connected feed-forward MLP to implement the visuomotor transformations for reaching toward the visual target. Figure 2C shows the schematic of the network architecture. The input to the MLP is comprised of 2 main types: (i) output of the CNN network that consists of extracted features from the input images, which has been flattened to facilitate further processing and (ii) extra-retinal signals, which can consist of several different signals such as eye position, head configuration, etc., but in the current implementation is restricted to eye position. These 2 types simply are concatenated for subsequent processing. The second layer is comprised of units that receive inputs from all the previous layer’s units and their activity is calculated as:

(11) \begin{equation*} {u}_j^l=f\left({\sum}_i{w}_{ij}^{l-1}\times{u}_i^{l-1}\right), \end{equation*}

where, ${u}_j^l$ is the activity of unit $j$ at the current layer $l$, ${w}_{ij}^{l-1}$ are learned connection weights from the $i\mathrm{th}$ to the$j\mathrm{th}$ unit, ${u}_i^{l-1}$ is the activity of unit $i$ at the previous layer, and $f(x)$ is the unit’s transfer function. In the current implementation, $l=2$. Here, we considered the sigmoid transfer function to mimic the nonlinear transfer function of real neurons (Naka and Rushton 1966) according to

(12) \begin{equation*} f(x)=\frac{1}{1+{\mathrm{e}}^{-x}}, \end{equation*}

The units in the third layer provide the population coding of the reaching movement. As described in Section Hidden units, these units are constrained to have cosine tuning. To impose such tuning behavior, we constrained the activity of the third layer’s units by fixing the connection weights to the final read-out layer according to equations (2)–(4). The final layer provides 2D effector displacement read-out of the population coded Euclidean distance. We first trained a separate feed-forward network with one hidden layer to transform the population codes into 2D read-outs. Then, we fixed the connection weights between the third and final layer. Units of the final layer are purely linear.

### Network training and testing

To provide realistic simulations and compare our model outputs with real data, we simulated the task employed in several previous experimental publications (Li et al. 2017; Bharmauria et al. 2020, 2021). As explained in Section Task, the task consists of 4 possible landmark locations and 8 possible landmark shifts. We extracted these landmark positions and their associated shifts as well as the target locations from the dataset. These data were used to create the encoding and decoding images. In addition, in this experiment, 3D eye movements (horizontal, vertical, and torsional components of orientation of the eye relative to space) were recorded (Bharmauria et al. 2020, 2021). For our simulations, we used the horizontal and vertical components to create the 2D initial and final gaze locations. We normalized final gazes by subtracting the mean gaze error calculated during no-shift condition. This normalization was done separately for each target location.

We made use of 2 types of datasets to train our network. First, a dataset was generated based on the available behavioral data obtained from monkeys (Bharmauria et al. 2020). We used ~25,000 trials for each of 2 monkeys, and we trained the network separately for each monkey. For each data point we had an encoding image (200 × 200), a decoding image (200 × 200), 2D initial eye position, and final gaze position to calculate the displacement of the eye. As described in the task, Section Task, an encoding image contains the initial eye fixation, target, and landmarks. Based on the task (Bharmauria et al. 2020), initial eye positions were distributed within 7°–12° from the center of the screen, whereas the target and landmark locations were determined based on the neuron’s response fields. Target locations were distributed approximately within a rectangular range of 30–80° across both horizontal and vertical dimension. Exact target locations were determined based on the size and shape of a neuron’s response field (i.e. 4 × 4 or 7 × 7 grid and 5–10° apart). Landmark locations were selected from one of the oblique locations 11° apart from the target. The decoding images contained the visual landmark: in the exact same location as the encoding image or shifted 8° from the initial location. The direction of the shift was randomly selected from the 8 possible locations evenly distributed on a circle (for a visualization, see Fig. 1).

To probe the network in more controlled, idealized conditions, we also generated simulated datasets for the combination of allocentric and egocentric information with 80,000 data points. We considered 3 scenarios: no allocentric (0% allocentric; final gaze location landed on the target location), purely allocentric (100% allocentric; final gaze location landed on the shifted target location), and a combination of allocentric and egocentric (30% allocentric; final gaze location landed between the target and shifted target location). Like the neurophysiological data, for each simulated data point we had an encoding image (200 × 200), a decoding image (200 × 200), 2D initial eye position, and final gaze position to calculate the displacement of the eye. For encoding images, we generated target and landmark locations randomly. These locations were uniformly distributed between (−40,40) and (−50,50) for landmark and target, respectively. For decoding images, landmark shifts were randomly chosen and varied in the range (−10,10). Similarly, initial gaze positions were generated randomly in the range (−10,10). All the values are generated in screen coordinates with (0,0) being the center of the screen.

We considered the mean square error (MSE) between a monkey’s final gaze displacement and the network output as the network loss for training,

(13) \begin{equation*} L= mi{n}_W\left({\sum}_{Tdata}{\left({x}_e-{x}_d\right)}^2+{\left({y}_e-{y}_d\right)}^2\right), \end{equation*}

where $W=\{{w}_1,\dots, {w}_N\}$ are the learned parameters, $Tdata$ is training data, $({x}_e,{y}_e)$ is network’s estimated gaze location, and $({x}_d,{y}_d)$ is ground truth gaze location. The numerical value for *N* is specified below for each layer.

The values for the predetermined parameters in the first 2 layers of the CNN were as follows: We used 4 Gabor filters (7 × 7) with uniformly distributed orientations: $\theta \in \{{0}^{{}^{\circ}},{45}^{{}^{\circ}},{90}^{{}^{\circ}},{135}^{{}^{\circ}}\}$. All the other parameters were the same for all the Gabor filters (i.e. $\sigma =1$, $\lambda =0.5$, $\gamma =2$, and $\psi =0$). We used a down-sampling of factor 2 for our spatial pooling layers. We used a sigmoid transfer function for the first layer of the feature pooling stage in the CNN (and the 2 first fully connected layers in our MLP). After the feature pooling, we flattened the images, which resulted in a vector with 4,136 × 1 dimension. We used 44 units to code initial gaze location and 100 units for our hidden layer. For our motor population codes, we varied the number of units from 4 to 250 and observed the same results. Here, we provided the results with 250 units. Overall, the learned parameters were 16 for the CNN and 13,391,200 for the MLP.

Training used the ADAM optimizer (Kingma and Ba 2015) with learning rate of 0.001 and batch size of 32. We divided each dataset into 3 sub-datasets: The first 2 subsets with proportion of 90 and 5 were used for training and cross-validation of the network. This separation was done to prevent overfitting. The final 5% was used for assessing the performance of the network against behavioral data. We used *R*^2^ to quantify the explained variance in the training dataset by our network and evaluate the network performance. We stopped the training based on 2 criteria: (i) if the number of epochs reached 50, or (ii) if the RMSE in the validation dataset stopped dropping significantly (i.e. absolute changes in gradients < 0.001). We provided our figures of our training in Supplementary Fig. S1.

### Hidden unit analysis

We deployed a similar analysis pipeline as Bharmauria et al. (2020) to analyze the activity of the hidden units in our network. This pipeline consists of 2 steps: (i) determining the coordinate frames that each unit uses to code information. The pool of possible coordinate frames is created either based on the canonical coordinates (e.g. Target in eye, shifted target in eye, etc.) or based on the intermediate coordinates (e.g. coordinates created by connecting target to the shifted target). (ii) Selecting units that are spatially relevant to the task. This selection is performed by examining if each unit’s response field is spatially tuned. In the following we elaborate on how we performed each of these steps.

#### Fitting units’ response fields against spatial models

A key step in understanding how the brain implements the required coordinate transformation is determining the intrinsic coordinate frame of individual neurons. To be consistent with the published neural literature (Bharmauria et al. 2020, 2021), we used the same analytic method, i.e. fitting different spatial models to a neuron’s response field (Keith et al. 2009). The spatial model that yields the best fit (lowest residuals between the data and model) is taken as representative of the intrinsic coordinate frame of the neuron. Further description is provided in the Supplementary Method section.

**Fig. 4:**
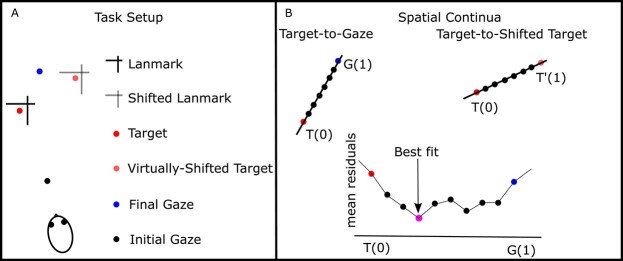
Spatial model fitting logic and procedure. A) Example of the task configuration: The box represents the real task under consideration, including both encoding (landmark and target) as well decoding (shifted landmark) images. B) Spatial continua: We selected 3 reference points to create spatial continua: Target, Gaze, and virtually shifted target locations. Using these 3 reference points we created 2 continua: Target-to-Gaze and Target-to-Virtually shifted Target. We performed model fitting across both continua and selected the model with the lowest mean residuals as the best model explaining intrinsic coordinate frames of the unit.

In our task, and based on the neurophysiological findings, we considered 3 main reference points (Fig. 4A): Target, final gaze, and shifted target locations. We also considered 2 possible coordinate frames, space vs. eye frames. To discriminate between these coordinate frames, we fit each unit’s response field against different spatial models (here 6: Target in eye (T_e_), Final gaze in eye (fG_e_), Shifted target in eye (T′_e_), Target in space (T_s_), Final gaze in space (FG_s_), and Shifted target in space (T′_s_)) using a nonparametric fit with a Gaussian kernel:

(14) \begin{equation*} {A}_{fit}\left({x}_i; kw\right)=\frac{\varSigma_{i\ne j}^n{A}_j\times{e}^{-{\left|{x}_i-{x}_j\right|}^2/ kw}}{\varSigma_{i\ne j}^n{e}^{-{\left|{x}_i-{x}_j\right|}^2/ kw}}, \end{equation*}

where ${A}_{fit}$ represents the fit prediction for a neuron’s activity, ${A}_i$ represents recorded activity for neuron $i$, $x$ represents the position, and $kw$ represents the Gaussian kernel bandwidth.

To quantify the goodness of the fit, predictive error sum of squares (PRESS; Keith et al. 2009) statistics were calculated. To do so, we calculated the fit for each trial based on the activity of the other trials. Then, we calculated error between the fit activity and the measure activity for each trial and calculated the mean squared difference for a given spatial model and kernel bandwidth. The model and kernel bandwidth that yielded the lowest residual is considered as the best fit. This kernel bandwidth is used for further analysis. Full details of this method are available elsewhere (Keith et al. 2009). Supplementary Fig. 3 provides further explanation of our fitting method.

#### Intermediate spatial models

Our previous results (Sadeh et al. 2015; Sajad et al. 2015, 2016; Bharmauria et al. 2020) suggested that canonical models (model’s that are merely based on different effectors and experiment parameters, e.g. Target in eye, shifted target in eye, etc.) are not always the best candidate to describe the neural response field, but instead intermediate models between canonical models should be considered. In addition, based on our previous observations (Bharmauria et al. 2020, 2021), we designed our motor population layer to code information in eye-coordinates. Consequently, in the following we only focus on the spatial models in eye-coordinates and will not use subscripting. Figure 4A shows a schematic of the geometric relation of the components of an example trial in the deployed task (explained in Section Task) and Fig. 4B demonstrates the 2 relevant continua. Therefore, we created 2 continua (Fig. 4B): Target-to-gaze (T–G), and Target-to-virtually shifted target (T–T′). We considered 30 equally distributed steps for each continuum (with 10 steps between the main reference points and 10 steps above each of the reference points). Furthermore, the rightmost panel in Fig. 4B shows the residuals for the 10 steps between the target and gaze continuum. The model that yields the lowest PRESS residual is selected as the best model fit.

**Fig. 5:**
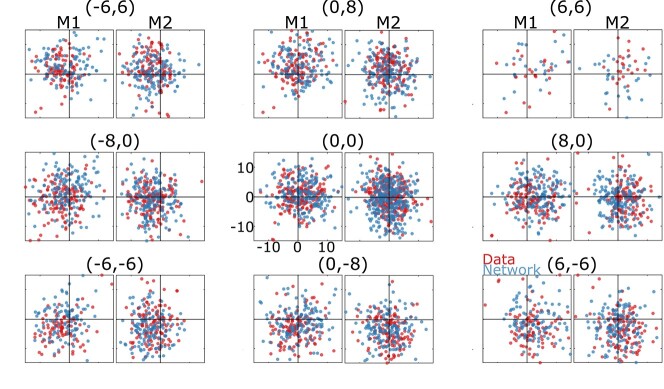
Comparison of 2-D Gaze end points generated by monkeys (red dots) versus model (blue dots) in the same task conditions. Data points are grouped into 9 panels based on the direction of the landmark shift. The amount of the landmark shift is shown on top of each figure, e.g. where (0,0) means no shift and (8,0) means an 8° horizontal shift to the right. Data were derived from many different target locations but here are normalized relative to target location (0°,0°). For each panel, the left plot represents the data for monkey 1 and the right plot represents the data for monkey 2. The large circles indicate the typical size (10°) of the monkeys’ reward window (range 8–12°). As illustrated, for both monkeys and our network, the final gaze distribution was centered around target location (and mostly within the reward window) but shifted partially in the direction of the landmark shift. In addition to the landmark influence, our data are affected by different sources of noise (e.g. sensory and motor noises).

One of the main assumptions here is that the landed gaze location will not be the same as the target location. This difference between gaze location and target is due to sensory, motor noises as well as the landmark shift (an illustration of the noise is provided in result Section Quantitative comparison between network  generated and behavioral gaze, Fig. 5). The T–G and T–T′ continua are selected to assess the intrinsic coordinate frames of neurons considering these sources of noise. The T–G continuum will provide the tool to evaluate the transition between target coding to motor performance. Particularly, as can be predicted, we observed that earlier during the task (e.g. target presentation) neurons code more target location and as the task progress (e.g. performing the saccade) neurons code gaze location. Similarly, the T–T′ continuum examines the effect of the landmark shift.

#### Test for spatial tuning

The method of Section Intermediate spatial models can be used only if a unit’s activity is spatially tuned, i.e. selective for a particular set of target positions. Therefore, we tested for spatial tuning of each unit’s activity and excluded the spatially untuned units from our analysis. To test for spatial tuning, we shuffled the average firing rate data over the position data obtained from the best-fitting model. Then, we statistically compared the mean PRESS residuals distribution of the 200 randomly generated response fields with the mean PRESS residual distribution of the best fit model. A neuron’s activity is considered spatially tuned if the best fit PRESS residual fell outside of the 95% confidence interval of the distribution of the randomly shuffled mean PRESS.

## Results

We designed and implemented an ANN and trained it on actual and synthesized visual stimuli/gaze data to model the neural integration of allocentric and egocentric cues. In the following sections, we evaluate this network in terms of both its behavioral (decoded gaze output) performance (Section Behavioral analysis: gaze output  performance and allocentric influence) and the unit and population properties of its MLP output layer (Section Neural analysis: MLP output response fields and  reference frames).

### Behavioral analysis: gaze output performance and allocentric influence

Figure 5 shows the distributions of gaze end points for both monkey data test sets (red dots) and the corresponding decoded model outputs for the identical initial position and stimulus inputs (blue dots). Data are normalized relative to target position but separated by landmark shift direction. The large circles indicate the typical reward window used during the monkey recordings (range 8–12°; Bharmauria et al. 2020). This results in 2 important qualitative observations: Both gaze datasets tend to cluster similarly around the targets (and mostly within the reward window), but both tend to shift subtly in the direction of the landmark shift. Overall, the actual and stimulated data distributions overlap, i.e. they follow the same general patterns.

To formally evaluate our network output, we tested it quantitatively against both actual gaze data (Fig. 5) and neural data recorded in the same task. Specifically, we addressed the following questions: First (Section  Quantitative comparison between network generated  and behavioral gaze), what proportion of the overall gaze variance can be replicated by our network? To answer this question, we evaluated overall network performance by comparing gaze endpoints and errors in model output to an equivalent monkey dataset with similar inputs, i.e. initial gaze, target, and landmark positions (Bharmauria et al. 2020). Second (Section Influence of allocentric  weighting and noise on overall gaze performance), how is overall gaze performance influenced by allocentric–egocentric weighting and noise in the training set? To answer this, we compared network performance with actual and simulated gaze datasets where we systematically manipulated allocentric influence and simulated noise in the training set. Third (Section Systematic  influence of the landmark shift on simulated gaze  output), does our network learn and replicate the observed allocentric–egocentric weighting? Here, we isolated the specific influence of the landmark shift relative to target position. We compared the allocentric–egocentric weighting produced by the network to see if it would replicate the weighting for different training sets (including actual monkey behavioral data).

#### Quantitative comparison between network generated and behavioral gaze

The goal of this test was to ascertain if the network learned the relevant aspects of the task. To this aim we evaluated if the network replicates the specific patterns and distributions of gaze behavior observed in monkeys on the same task. Here, we used the data of the 2 monkeys (Bharmauria et al. 2020) to train and evaluate network performance. Note that for these tests we compared monkey gaze data with gaze positions decoded from the MLP output layer of our model under identical conditions (initial gaze, target position, and landmark stimuli). We tested the model on portions of data that were not used during training or validation.

**Fig. 6:**
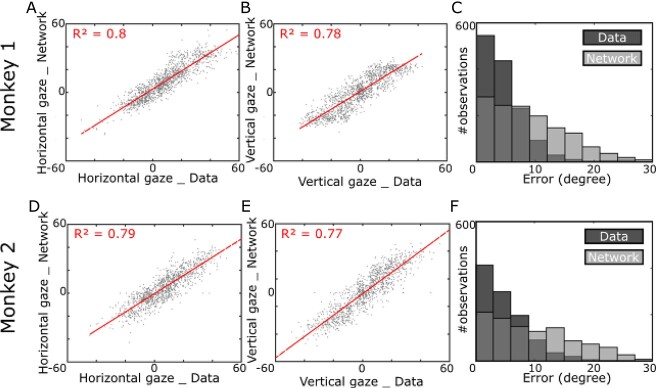
Quantitative analysis of overall network performance B). A–B) Regression analysis of our network gaze endpoints and the monkey gaze end points. Our network explained approximately 80% of the data’s variability for both horizontal and vertical directions. C) Distribution of gaze endpoint errors observed from the monkey data (darker gray boxes) and from our network (lighter gray boxes). For both monkey data and our network, the errors are mainly below 8–12°. D–F) similar analysis as A–C) but for the second monkey. Note that the overlap between the 2 bar plot results in the medium dark gray.

To evaluate how well our network learned the task, we calculated the goodness of fit of the network output to the actual gaze displacement data using *R*^2^, which assesses the ability of our network to explain the data’s variability (Fig. 6A–B and D–E). Broken down into horizontal and vertical components, the model explained 80% of the horizontal variance and 78% of the vertical variance for the first animal and 79% of horizontal and 77% of the vertical variance for the second animal. Overall, our network explained 80% of the data’s end-point variability in the first animal, and 75% in the second animal (Table 1, rows 1–2). This is likely due (mostly) to their dependence on target location, but possibly also due to other factors that we will examine below.

**Table 1:** Evaluation of our network performance under different scenarios and comparison to monkey data. The first 2 rows present the result for actual data and the 4 final rows present the result for the simulated data with different allocentric–egocentric combination and noise values.

Scenario		Testing	Training		
Allocentric contribution	Noise	*R^2^*	MSE	# Training points	# Testing points
Monkey 1	NA	0.80	58.22	25,626	1,424
Monkey 2	NA	0.75	63.04	21,638	1,202
0%	No noise	0.93	39.79	64,000	8,000
100%	No noise	0.95	56.82	64,000	8,000
30%	No noise	0.94	51.96	64,000	8,000
30%	High^*^	0.87	115.19	64,000	8,000

We chose the noise to simulated the observed variability in the data: (random gaze variations introduced by adding Poisson noise as well as Gaussian blur with 10deg standard deviation for images)

In addition, we asked how similar the distribution of the gaze error amplitudes is (calculated as the angular distance between the end gaze point and target position) in our network compared with the data. Figure 6C and F illustrates the comparison of the error distribution for our network (light colors) and the monkey behavior (darker color). As can be seen, monkey data were very noisy, but the majority of the errors fell below 8°–12° of the visual target. In the behavioral task, monkeys were rewarded if they performed a saccade within 8°–12° distance of the target (Bharmauria et al. 2020). This reward window was selected to reassure that monkeys were engaged with the task, but it is not a criterion for data exclusion in our model. Qualitatively, the trained network errors follow a similar distribution, except errors dropped off less precipitously outside the monkey’s reward window.

These results suggest that (i) our model explains the majority of the observed gaze behavior variability and (ii) that the network and monkey data showed similar distributions of gaze error, with most gaze trials landing near the target. Thus, most of the explained variability is likely due to target influence, but it is possible that some of the explained variability is due to the landmark shift (see Section Systematic influence of the landmark shift on  simulated gaze output). But first, we will assess how well the model performs on other training sets, and the possible role of the noise in the unexplained variability of these fits.

#### Influence of allocentric weighting and noise on overall gaze performance

The purpose of this analysis was to evaluate how overall model performance (i.e. gaze accuracy, precision) is influence by (i) allocentric weighting and (ii) noise in the training set. For the first goal, we created 3 different synthetic training sets, with different levels of allocentric–egocentric weighting: 100%–0%, 0%–100%, and 30%–70% (similar to that observed in experimental studies). In other words, the final gaze positions were located on the target, on the virtual shifted target, or 30% shifted toward the virtual shifted target respectively, in the training set. No noise was present in these datasets. Table 1 (rows 3–5) summarize the network’s performance for these scenarios, providing *R*^2^ values for ideal vs. actual performance (Note that for this test, different datapoints were used than those used in the training set). It is seen that all 3 task situations, the network is capable of predicting a considerable amount of the data’s variability (>90%).

The performance of the model was better in these idealized datasets compared with the performance of the model trained on actual data. We hypothesized that this was very likely because there was noise in the monkey data unrelated to the task. To test this hypothesis, we created a fourth synthetic training set: again 30%–70% allocentric–egocentric, but with added noise (random gaze variations introduced by adding Poisson noise as well as Gaussian blur with 10^°^ standard deviation for images) similar to that seen in the data. This manipulation decreased the explained variability from 95% to 87% (Table 1, row 6), supporting the notion that much of the unexplained variance in our data-trained model was due to input noise rather than some failure of the model to simulate systematic behavior.

In summary, these initial tests suggest that the model output generally simulated monkey gaze performance in terms of end points and errors (Fig. 5) and that much of the disagreement between them is likely due to noise that cannot be trained into the network (Fig. 6). However, we have not yet established the specific contribution of the landmark shift to gaze errors.

#### Systematic influence of the landmark shift on simulated gaze output

The purpose of this section is to evaluate the specific influence of the landmark shift on allocentric–egocentric weighting in our network output. The key feature of allocentric–egocentric integration in human and monkey behavioral studies is that when these cues conflict, humans and monkeys perform as if weighting between them. The weighting of allocentric cues tested so far has ranged from 30% to 50%, depending on the experimental conditions (Neggers et al. 2005; Byrne and Crawford 2010; Fiehler et al. 2014; Klinghammer et al. 2017; Li et al. 2017; Lu and Fiehler 2020). This result presumably reflects an optimization process, where usually egocentric and allocentric cues would tend to agree with each other, but with different levels of reliability and noise (Byrne et al. 2007; Körding et al. 2007; Byrne and Crawford 2010; Lew and Vul 2015; Klinghammer et al. 2017; Aagten-Murphy and Bays 2019). Here, we directly tested if our network learned to perform such integration.

To answer this question, we assessed whether the gaze endpoints produced by our trained networks replicate the influence of the landmark shift when actual monkey data are used in the training set. The influence of the shift is quantified by the component of the gaze endpoints (d) along the axis between the target (T) and direction of the landmark shift (T′) (Bharmauria et al. 2020). As this parameter quantifies the contribution of allocentric information in reaching, it is taken as the allocentric weight (AW): AW = 0 means that no allocentric information is used (i.e. the gaze endpoints were in close vicinity of the memorized target), whereas AW = 1 means that gaze is completely influenced by allocentric information (i.e. gaze endpoint shifted with the same amplitude and direction as landmark shifts).

**Fig. 7:**
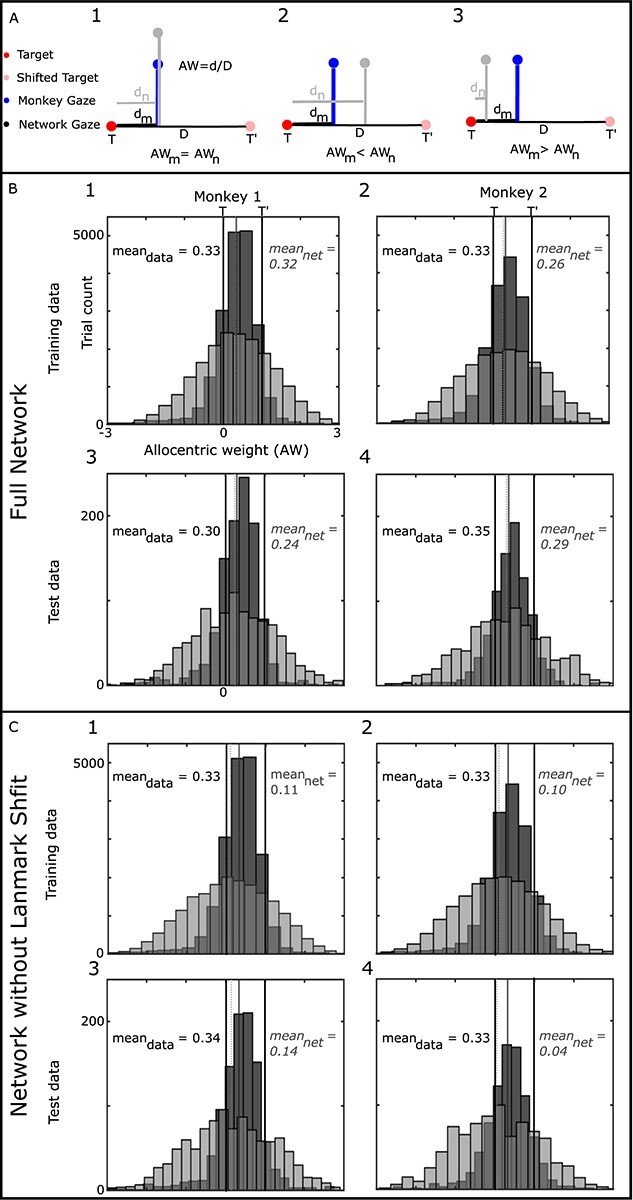
Allocentric weight based on Monkey’s behavior. A) Comparison of the network error vs. allocentric weights. In all panels the network error (predicted gaze (gray dot)—monkey gaze (dark pink dot)) remains the same; however, each configuration results in a different allocentric weight. This shows that solely training the network on the final gaze behavior does not guarantee the effect of allocentric information. B) Comparison of allocentric weights from our network vs. monkey behavior. As can be seen for both monkeys, the network replicates the observed allocentric weight for the training data set (shows proper training of our network) as well the test data set. C) Similar analysis as B but without providing landmark shift for the network. Although both networks (with and without landmark shift) yielded comparable errors, as opposed to the full network, removing the landmark shift from the network resulted in a poor replication of the allocentric weights for the training and test datasets.

Figure 7A illustrates the relationship between AW and task components. It is noteworthy that training the network on the gaze behavior does not guarantee that the network will replicate the observed AW distribution. This is shown in different panels of Fig. 7A: In all 3 panels, the returning value from loss function is the same (calculated as the difference between the network generated gaze (light gray dot) and gaze from data (blue dot); however, the AW generated by the network is different in all the examples. Thus, a network optimized to follow the gaze behavior does not guarantee the replication of the AW distribution.

To directly compare our network’s prediction with monkey’s behavior, we trained the network on each animal’s dataset and then compared AW of our network and the model for both the training datasets (Fig. 7B 1–2) and the test dataset (Fig. 7B 3–4). We used both training and test dataset to evaluate the training and prediction capability of our network. Notably, in all cases (both training sets and test sets) the gaze endpoints are shifted toward the shifted landmark with average AW ranging in [0.30–0.35] and [0.24–0.32] for the data and network, respectively. To quantitatively examine if the generated mean of the AWs is significantly different from the data, we used Wilcoxon signed rank test to compare the mean of the 2 distributions. Except for training set for the first monkey (*P* = 4.3124e−16), we found that the generated AW mean by our network is not significantly different form the monkey data (monkey 1 test set: *P* = 0.096; monkey 2 training set: *P* = 0.341; and monkey 2 test set: *P* = 0.0284). This result indicates that our network learned the fundamental aspect of the task (i.e. allocentric–egocentric combination).

To verify that these results are specific to the task-related inputs, we repeated the same simulations but removed the landmark shifts from the input (the decoding images was set to zero). Although the goodness of the fit for this modified network was 75% (very similar to the full model), the averaged AW for both training and test dataset were reduced to [0.10–0.11] and [0.04–0.14] for models trained on data from animals 1 and 2, respectively. These values were significantly different compared with the monkey behavior (Fig. 6C; monkey 1 training set: *P* = 0; monkey 1 test set: *P* = 2.2475e−16; monkey 2 training set: *P* = 0; monkey 2 test set: *P* = 6.0696e−10). In other words, the complete model with landmark-shift inputs was required to replicate the monkey behavior.

**Fig. 8:**
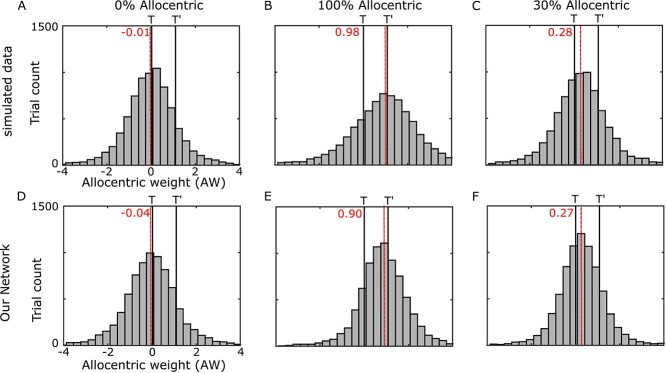
Allocentric weight based on simulated datasets. A–C) Allocentric weights for different simulated datasets. A) No allocentric contribution: The final gaze location landed on the remembered target location, B) full allocentric contribution: The final gaze location landed on the virtual target location, C) partial allocentric contribution (30%): Final gaze location landed between the remembered target and virtual target location. The percentage determines the amount of shift toward the virtual target. D–F) Our network’s replication of the final gaze locations for different simulated datasets where the differences arise from different selected experiment parameters (i.e. allocentric weight and noise). In all scenarios, the network was able to adapt the location of the final gaze location based on the desired allocentric contribution (0%, 100%, and 30%).

Finally, to show directly that our network can learn arbitrary allocentric–egocentric weightings, we repeated this analysis on our synthetic datasets with 0%, 100%, 30% allocentric weighting, with noise included to resemble the actual data (see Section Influence of  allocentric weighting and noise on overall gaze perfor-  mance). Figure 8 illustrates that our network learned different integration of allocentric and egocentric information dependent on the training dataset. For instance, varying the contribution of allocentric information from 0% (Fig. 8A) to 100% (Fig. 8B) resulted in a shift of the gaze endpoint distribution from being centered around 0 (Fig. 8D) to being centered ~1 (Fig. 8E). Similarly, a partial integration of allocentric–egocentric information resulted in a partial shift of gaze endpoint distribution (Fig. 8C vs. Fig. 8F). Based on these simulations, we conclude that the model is able of achieving arbitrary levels of allocentric–egocentric integration, depending on the input.

### Neural analysis: MLP output response fields and reference frames

We aimed to produce a model that can be compared directly with observed neurophysiological data. Specifically, we tested if the model was able to recreate a similar distribution of spatial coding observed in actual FEF data in the same task (e.g. Sajad et al. 2015; Bharmauria et al. 2020). Here, the key observations were that (i) FEF motor responses code gaze in an eye-centered frame of reference, (ii) this code was partially shifted toward the shifted landmark, and (iii) the influence of the landmark shift was fully integrated into the eye-centered motor responses of saccade.

To assess if our network shows similar results, we created a simulation of the neurophysiology experiments. To provide a uniform dataset of target/landmark combinations we generated a simulated training/testing dataset that contained a uniform distribution of target locations across the encoding-decoding images. This latter design was essential to ensure that we have enough units responsive to different target locations. (Note that our actual behavioral data missed many of these points, but networks trained on the behavioral data gave similar results to those described below.) To generate the final gaze positions, we created a Gaussian distribution for egocentric (Target) and allocentric (Virtually shifted target) information. We then created a distribution for the final gaze position using a weighted summation of allocentric and egocentric information (Bayesian integration; Byrne et al. 2007; Körding et al. 2007; Klinghammer et al. 2017). For our integration, we considered 33% weights for the allocentric information. This weight selection resulted in a similar distribution to the observed data (as shown in the previous section). We also added noise to replicate the monkey data (similar to row 6 in Table 1). Finally, we sampled from this distribution to generate the gaze position for each trial. We used this dataset to train our network. Then, we created a dataset where target location incrementally changes across the images to assess the intrinsic coordinate frames of hidden units in our motor population layer. We defined the motor population layer to resemble the FEF code (Bharmauria et al. 2020, 2021), as explained in the methods. For these simulations, we simulated 250 MLP output neurons with 2D directional tuning distributed evenly across 360°. Using these datasets, we compared general response field and reference frame properties (Section Response fields and intrinsic coordinate frames  of MLP output units), the influence of the landmark shift on both response fields (Section Influence of  landmark shift on MLP output unit response fields) and reference frame coding (Section Influence of landmark  shift on intrinsic coordinate frames of MLP output units), and finally the degree of integration between egocentric and allocentric coding in these units (Section Integrated  landmark influence in the final motor response).

To remind the reader, we analyzed the intrinsic coordinate frames of our units similar to the performed neural analysis in Bharmauria et al. (2020): First for each neuron we examined which coordinate frames it used to code information (e.g. target, gaze, landmark, shifted landmark, etc.), then we created intermediate coordinate frames to examine the egocentric vs. allocentric coding. Like the neural data, we created 2 continua: target to gaze (T–G) and Target to shifted target (T–T’). Full details of our neural analysis are provided in Section Hidden unit analysis.

#### Response fields and intrinsic coordinate frames of MLP output units

Recall that we constrained our output to represent FEF motor neurons. To this aim, we implicitly forced the motor population units to have cosine tunings with open-ended response fields. Here, we first confirm that our motor units behave as they are designed. Figure 8A shows an example motor response field, from a unit in motor population layer, in the absence of a landmark shift. The plotting conventions are the same as those used for real neural data in our previous papers, i.e. circle size indicates the neural “firing rate” for a randomly selected subset of the individual trials, and the color code represents the nonparametric fit made to the full dataset. Both the individual trial locations and fit are plotted in the coordinate system that provided the best overall fit for this unit: future gaze relative to initial eye position (see methods and below for further explanation). Similar to our actual FEF motor response recordings (Sajad et al. 2015; Bharmauria et al. 2021), we observed that only a subset of our neurons is spatially tuned (similar to Bharmauria et al. 2021) and that spatially tuned units showed open-ended response field (as expected).

**Fig. 9:**
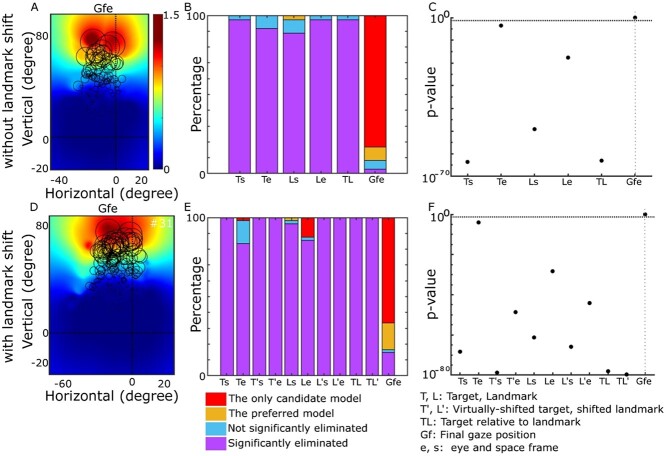
Intrinsic coordinate frame analysis for motor population’s units. A–C) without landmark shift: A) The response field of a motor unit. This unit has an open-ended response field with and upward preference. Cooler colors represent lower activity and warmer colors represent higher activity. Our nonparametric fit revealed that this unit significantly (*P* < 0.05) prefers a gaze-in-eye coordinate frame. The prediction of our fit is presented by black circles where the diameter indicates the activity predicted by our fit. We reduced the number of circles (randomly) for illustration purposes. B) Summary of the preferred coordinate frames for all 34 spatially tuned motor neurons. The tested models were Target in space (Ts), Target in eye (Te), Landmark in space (Ls), Landmark in eye (Le), Target in landmark (TL), and Gaze in future eye (Gfe). The majority of neurons (~83%) significantly (*P* < 0.05) preferred gaze-in-eye coordinate frames. C) Population analysis of units coordinates frames. The population analyses were performed by a *t*-test of mean residuals for each model relative to the best model fit. When coordinate frames were tested at the population level, the entire population significantly (*P* < 0.05) preferred gaze-in-eye coordinates. D–F) Similar analysis as A–C) but in the presence of the landmark shift. Here we had 48 spatially tuned neurons. In addition to the previously mentioned spatial models, models related to landmark shifts were tested: Virtually-shifted target in space (T′s), virtually shifted target in eye (T′e), shifted landmark in space (L′s), shifted landmark in eye (T′e), and Target in shifted landmark (TL′).

The next step is to investigate the coordinate frames our units deploy to code information. Although the overall population code follows an eye centric coordinate frame (Gfe) as an emergent result of our design, the individual neurons can have different coordinate frames. To test the intrinsic coordinates of our simulated neural population, we used the same methods used in our previous physiological studies (Bharmauria et al. 2020, 2021). In brief, we calculated the residuals between the individual data points and fits made in each coordinate system. Only units that showed significant spatial tuning were selected for further analysis (*n* = 34 in the absence of landmark shift). We tested Target-in-space (Ts), Target-in-eye (Te), Landmark-in-space (Ls), Landmark-in-eye (Le), Target-relative-to-landmark (TL), and future Gaze relative to initial eye position (Gfe). We found that the majority of individual units (~83%) showed a significant preference for Gfe coordinates, and no neurons showed a significant preference for the other models (Fig. 9B). Similarly, the entire population showed a significant (*P* < 0.05) preference for gaze-in-eye coordinates (as expected; Fig. 9C), similar to Sajad et al. (2015), where there was no landmark.

We repeated the same analysis in the presence of the landmark shift (Fig. 9D–F), where 48 of the 100 output units showed significant spatial tuning. Here, additional models were included to account for the shifted landmark position (L′) and the virtual position of the target relative to the landmark (T′L′). However, these results showed a similar pattern: Response fields were very similar (Fig. 9D), a few units showed a significant preference for Le but the majority (~67%) showed a significant preference for Gfe (Fig. 9E) and the entire population significantly preferring future gaze-in-eye coordinates (Fig. 9F). In short, these results replicated those recorded from the monkey FEF in the presence of a landmark shift: preservation of the basic eye-centered gaze code (Bharmauria et al. 2020). But as in the latter study, this comparison of “canonical” models was not sensitive enough to detect the influence of the landmark shift. For that, we turned to a more sensitive analysis based on intermediate coordinate frames, as shown below.

#### Influence of landmark shift on MLP output unit response fields

**Fig. 10:**
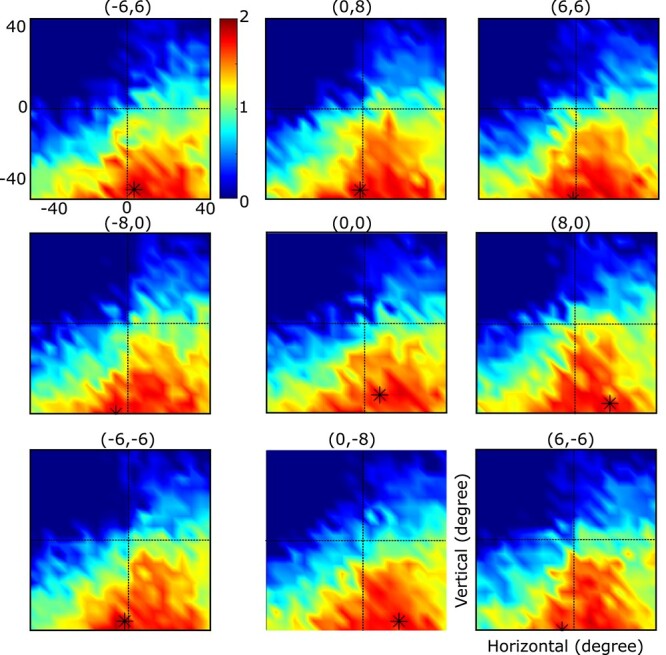
A unit’s response field for different landmark shifts. Each panel shows the response field of the same unit for different landmark shifts. The coordinates of landmark shifts are provided on top of each box (in parenthesis). Response fields are plotted in gaze coordinates. The peak of activity is indicated by an asterisk (^*^) in each box. Following the asterisk location, varying the landmark shift resulted in a correspondingly shifted response field in some directions indicating the possibility of shifted intrinsic coordinate frames.

In monkeys, FEF motor responses are modulated by a shift in the landmark, specifically causing a shift toward landmark-centered coding without altering the basic response field or gaze code (Bharmauria et al. 2020). To see if a landmark shift produced a similar influence in our simulated data, we first plotted unit response fields for different landmark shifts. A typical example is shown in Fig. 10. Neural activity is represented by the heat map, with an asterisk (^*^) placed at the peak of the response field, plotted in Gfe coordinates and indicate the coordinates of landmark shifts on top of each box (in parentheses). The middle panel in Fig. 10 shows the response field for no-shift condition (0,0), and the other panels are arranged congruently with the landmark shift direction (i.e. up for up-shift etc.). Again, the unit activity resembles an open-ended response field. At first glance, the Gfe response field appears to be very similar for each landmark shift, consistent with the notion that this unit is primarily coding gaze. However, the peak of the response field appears to shift with the landmark in some directions (e.g. for left and right shifts in this case) but not other shift directions. This result was typical, but other units showed direction-dependencies (of the cue shift) on both the magnitude and direction of their response field shifts (see Supplementary Fig. S2). To understand the overall landmark influence on this population, we re-examined the underlying coordinates of these response fields, using a more sensitive test (Bharmauria et al. 2020, 2021).

#### Influence of landmark shift on intrinsic coordinate frames of MLP output units

In the next step, we performed a similar “intermediate frame” analysis as used in our previous neurophysiology studies (e.g. Bharmauria et al. 2020). Specifically, we used nonparametric fits (as described in Section Hidden unit analysis) to detect the best fits for each spatially tuned unit along 2 spatial continua: Target to shifted target (T–T′) and Target to Gaze (T–G). T–T′ provides a continuous measure of the influence of the landmark shift on the target representation, where T′ would be a virtual target fixed to the shifted landmark (Bharmauria et al. 2020). T–G measures the degree to which each unit encodes variable gaze errors (Sajad et al. 2015). Each continuum was divided into 10 steps between the 2 “cardinal” models with 10 more steps beyond each. The point yielding the lowest fit residuals represents the best intrinsic coordinate frame.

**Fig. 11:**
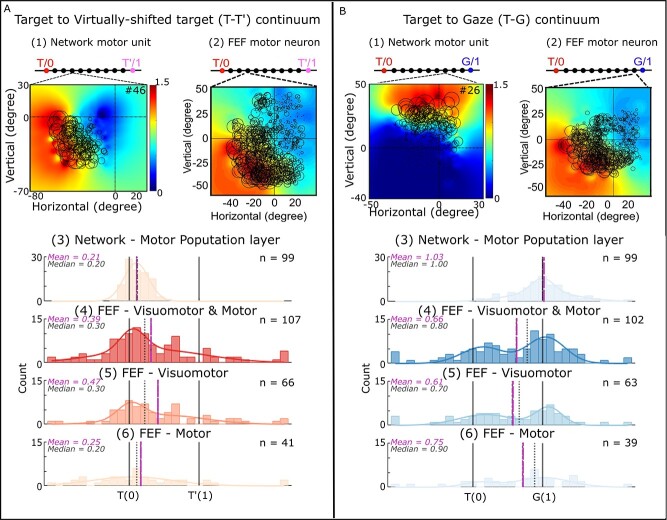
Motor neuron’s coordinate frames. Model hidden units at the motor population layer showed similar coding mechanisms as the neurophysiology data. A) Model fits in allocentric coordinates (i.e. along T–T′ continuum). 1–2) Fit model for a sample unit in our motor population layer (1) and in FEF (2). In both example neurons, the intrinsic coordinate frame was 3 steps away from the target toward the shifted target. 3–6) Distribution of the intrinsic coordinate frames when fit along T–T′ continuum. Motor population coding in our network (3) was biased toward target coordinates when fit along the Target-to-Shifted target coordinates with a partial shift toward the shifted target (30%). This shift was aligned with the distribution of the FEF neurons (4–6). Separating the different classes of neurons (visuomotor (5) and motor (6)) showed that our motor population neurons resemble mostly the motor only neurons (as expected). B) Model fits in egocentric coordinates (i.e. along T–G continuum). 1) Fit model for a unit in our motor population layer. 2) Fit model for an example neuron. In this neuron the best model is 1 step before the final gaze model. 3–6) Distribution of the intrinsic coordinate frames of motor neurons in our motor population layer and area FEF (Bharmauria et al. 2020). 3) The majority of motor units coded information in Gaze coordinates. 4–6) Similar to our simulated motor units, the majority of neurons coded information in Gaze coordinates. However, in the physiological data, the fits represent a bimodal distribution as opposed to a unimodal distribution. Separation of the visuomotor neurons (5) from motor neurons (6) showed that most of the bimodality is resulted from the visuomotor neurons. This observation suggest that our motor units mostly represent the motor only neurons and not the motor response of the visuomotor neurons.

Figure 11 provides a direct comparison of our network’s motor population layer vs. actual FEF motor responses. Figure 11 A show fits along T–T′ continuum. The first figure (Fig. 11A (1)) shows an example of a simulated motor unit that codes information in an intermediate coordinate frames that is shifted 3 steps in the direction of T′ (Landmark shift), with a very similar FEF neuron (also shifted 3 steps) illustrated in Fig. 11A (2). The rows below (Fig. 11A (3)–(6)) show corresponding frequency histograms for the simulated and physiological data. The distribution of the simulated fits was narrower compared with the physiological data fits but they both show a shift to the right, i.e. in the direction T′. The simulated data (Fig. 11A (3)) showed ~20% shift toward the T′ (mean = 21%, median = 20%, *P* = 7.54e−7, Wilcoxon signed ranked test). The distribution of the fits for simulated data followed a Gaussian distribution. For the physiological data (Fig. 11 (4)) the shift had a mean of 29% (median 20%) toward T′. In our physiological studies (Sajad et al. 2015; Bharmauria et al. 2020), 2 types of neurons were presented: visuomotor neurons (neurons that are responding both at the time of visual stimuli and eye movement) and motor neurons (neurons that are responding only at the time of eye movement). To further compare our network’s motor population units’ behavior with FEF neurons, we separated the visuomotor neurons from motor neurons (Fig. 11 (5) & (6)). We observed that the visuomotor neurons (Fig. 11 (5)) show a bimodal distribution (resembling a mixture of Gaussians) in their fit with higher shift toward T′, whereas motor neurons (Fig. 11 (6)) show a unimodal distribution with lower shift toward the right direction. This comparison suggest that our motor population units resemble to motor neurons and not the visuomotor neurons. In addition, these observed shifts, along the T–T′ continuum, are qualitatively consistent with the observed 33% allocentric shift in the behavioral data (see Section Systematic influence of the landmark shift on simulated gaze output). Overall, these results suggest that, like the actual data, our MLP output unit coordinates shifted partially with the landmark toward coding T′.

Figure 11B provide a similar analysis for the T–G continuum. The example response fields (Fig. 11B (1) & (2)) were shifted 70% and 90% from T toward G in the simulated and FEF data respectively. The rows below (Fig. 11B (3)–(6)) show the distribution of best fits for the simulated and physiological data. The simulated data (Fig. 11B (3)) showed a unimodal distribution with a significant shift (*P* = 1.06e−18, Wilcoxon signed ranked test) toward G that was larger on average with a mean of 100% and median of 100%. The FEF data (Fig. 11B (4)) showed a bimodal distribution with a smaller peak near T and a larger peak near G, and an overall mean and median of 57% and 70%, respectively. Separating the visuomotor and motor neurons, we observed that the majority of the bimodality resulted from the visuomotor neurons (Fig. 11B (5)) whereas the distribution of the motor neurons (Fig. 11B (6)) was less bimodal and had a higher shift toward G. Nevertheless, the dominant peaks of all datasets (simulated and physiological) indicated near pure gaze coding.

Overall, these results indicate that the model, like most actual FEF responses, show a coordinate shift in the direction of the landmark while continuing to code gaze relative to initial eye orientation. Although this might sound contradictory, in the next section we show how these 2 results can be reconciled.

#### Integrated landmark influence in the final motor response

**Fig. 12:**
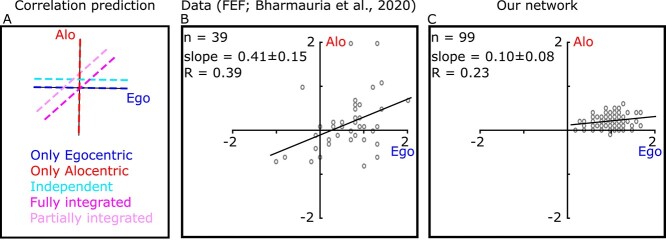
Integration of allocentric and egocentric information. A) Correlation prediction for different allocentric and egocentric integration scenarios. B) Allocentric information was integrated into FEF motor codes. When allocentric coding is examined against egocentric coding, it showed significant correlation. C) Similar to the FEF data (Bharmauria et al. 2020), our motor population layer showed integrated allocentric information into the motor codes (significant correlation between allocentric and egocentric coding; slope = 0.10 ± 0.08, *R*^2^ = 0.1, *P* = 0.0200).

In the previous section, we showed that similar to FEF motor neurons, our motor population units code information in both egocentric (T–G) and allocentric coordinates (T–T′). An important question is whether these 2 codes are integrated or independent. To answer this question, we correlated our allocentric and egocentric codes. As illustrated in Fig. 12A, these codes could be completely independent (vertical or horizontal lines), multiplexed but uncorrelated (shifted lines), or correlated (diagonal lines). In the physiological data recorded previously (Bharmauria et al. 2020), these measures were uncorrelated in memory responses (not shown), but became significantly correlated in the motor response, suggesting an integrated motor code (Fig. 12B). We tested if the same was true in our simulated data. Although the simulated data had a smaller distribution, we observed a significant ego-allocentric correlation (Fig. 12C; slope = 0.10 ± 0.08, *R*^2^ = 0.10, *P* = 0.0200) in our motor output layer. This result suggests that our network replicates the observed behavior in monkeys FEF motor responses, i.e. an allocentric influence that is integrated into the egocentric motor response to directly influence gaze behavior.

## Discussion

In this study, we developed and validated a novel neural network framework of allocentric–egocentric integration for goal-directed movements. The model used physiologically constrained inputs and outputs and was validated through comparison of (i) network output with known monkey gaze behavior in cue-conflict task and (ii) its hidden unit’s activity with FEF neural activity recorded in this same task (Bharmauria et al. 2020). To implement a general theoretical framework, we modeled the visual system as an analytically specified CNN and the sensorimotor transformation for gaze as an MLP. A pretrained decoder was added at the end of the MLP to transform the motor population activity into 2D saccades. The network (i.e. MLP weights) was trained on synthetic and actual data (final gaze locations) from a task where a landmark shifted relative to the saccade target. We observed that (i) our network generates saccade vectors comparable to observed monkey gaze as well as simulated datasets with different allocentric–egocentric contribution, with and without noise, (ii) the network replicates the allocentric–egocentric weighting in different datasets, and (iii) our motor units code gaze in an eye-centered frame (an emergent property of our network design); this code was partially shifted toward the shifted landmark and the influence of the landmark shift was fully integrated into the eye-centered motor responses of saccade (result of our network training), similar to FEF motor neuron responses. To our knowledge, this is the first network model that combines complex visual system properties with a sensorimotor transformation to replicate observed behavioral and neural data.

### Neural network models in sensorimotor neuroscience

Neural network approaches have long been deployed to understand the underlying mechanisms for sensorimotor transformations (for reviews, see Pouget and Snyder 2000; Battaglia-Mayer and Caminiti 2018; Blohm et al. 2009). The general approach is to train an analogous network using similar input–output as observed in sensorimotor tasks, evaluate network output and performance against experimental data (as we have done here), and then analyze hidden unit properties to understand how the brain might do this. Early studies modeling sensorimotor transformation in 2D found that varying initial eye, head, and hand positions results in gain modulation of unit’s activity (Zipser and Andersen 1988; Salinas and Abbott 1995; Xing and Andersen 2000). Analysis of hidden layers revealed that hidden units encode information in purely gaze-centered coordinate frames, whereas the information is coded in intermediate coordinate frames or as shifting response fields in motor output layers. Extended from 2D modeling, a 3D neural network investigation revealed new properties generalizing the previous findings (Smith and Crawford 2005; Blohm et al. 2009). The authors observed fixed input–output relationships within each unit and each layer. These fixed input–output relationships acted as local coordinate transformation modules. The global transformation at the network level was implemented by combining these local transformations, weighted in a gain-field like fashion (Blohm et al. 2009). These new properties emerged as a result of the nonlinearities inherited form the 3D coordinate transformations. Other studies have extended such models to include recurrent connections, “memory” and spatial updating of the remembered visual goal during eye movement (Keith et al. 2010).

The above-mentioned models played a crucial role in understanding the available data as well as providing prediction for further experiments. However, these studies have 2 shortcomings. First, in all the previous studies, the complexity of visual system is ignored: The visual stimulus is oversimplified into a “dot” that is represented as a hill of activity in a retinal topographic map. This oversimplification results in the inability of such networks to model complex stimuli (e.g. crossing lines, or objects). Thus, previous models are not capable of explaining the presentation of allocentric information and their role in sensorimotor transformations. Second, the majority of the previous studies treated sensorimotor transformations as a feedforward network that resulted in a lack of temporal dynamics. Here, we attempted a first step toward addressing the first of these 2 limitations (visual complexity), as discussed in the following sections.

### Current approach

To create and validate a model that would be useful for neurophysiologists, we followed principles established in previous studies described previously (Zipser and Andersen 1988; Blohm et al. 2009; Keith et al. 2010). In particular, we constrained the inputs and outputs of our network to resemble the known physiologically of the modeled system. Since our aim was to reconstruct the early visuomotor transformations from visual cortex to early 2-dimensional (2D) eye-centered motor codes in frontal cortex (Bharmauria et al. 2020, 2021), we simplified the inputs as 2D eye-centered images and 2D eye-position signals based on the eye-position coding observed in Somatosensory area 1 (S1) (Wang et al. 2007). Likewise, we modeled the output layer to represent the open-ended response fields observed in the FEF (Bruce and Goldberg 1985; Sommer and Wurtz 2006; Sajad et al. 2015, 2016). This particular input–output configuration allowed us to both train and compare our model outputs with a known dataset (Bharmauria et al. 2020, 2021).

In contrast to previous visuomotor networks (which processed a single “dot” stimulus), our model had to encode a 2D visual image containing multiple features and then somehow represent/integrate egocentric and allocentric information derived from this image. To do this, it was necessary to combine the complexity of the visual system (Schenk 2006; Thaler and Goodale 2011; Geirhos et al. 2017; Rajalingham et al. 2018) with the feed-forward properties of a sensorimotor transformation (Smith and Crawford 2005; Blohm et al. 2009; Crawford et al. 2011). For this purpose, we found it useful to separate the network into a representational stage (i.e. the visual system) and a sensorimotor transformation stage (Crawford et al. 2011).

To model the visual system, we used a CNN. But unlike previous models (Geirhos et al. 2017; Rajalingham et al. 2018; Schrimpf et al. 2018; Kar et al. 2019; Lindsay 2021) we did not train our CNN but instead used an analytically constrained architecture to be consistent with the known physiology. Specifically, we built our CNN based on the concept of repeated filtering: “filter-rectify-filter.” Our network consisted of 2 identically designed layers in which each layer consists of convolution, rectification, normalization, and pooling. To replicate the properties of cortical simple and complex cell, we deployed 2D oriented Gabor filters for our convolutions (Carandini et al. 2005). Following the biological evidence suggesting that learning occurs at higher cortical levels (Serre et al. 2005), we introduced a trainable feature pooling layer to construct the final output from the CNN with the required attributes (here landmark and visual target). This design enabled us to construct a highly interpretable model of the visual system. In particular, the model is interpretable in that each stage admits to precise mathematical description, as given by the equations of Section Convolutional neural network.

To model the sensorimotor system, we used the outputs of our CNN as inputs for a fully connected feedforward MLP. In line with sensorimotor transformations studies, this network was left fully trainable. As noted above, previous studies have shown that such feedforward networks are able to produce striking similarities with observed neural activities in areas involved in sensorimotor transformations (Zipser and Andersen 1988; Blohm et al. 2009; Keith et al. 2010). For this purpose, it is advantageous that our network is highly interpretable, i.e. it is not a black box as is often the case for many ANNs.

Finally, we trained and validated our model against real data. We trained our network using both idealized datasets and behavioral data from a recent neurophysiology experiment (Bharmauria et al. 2020). For training purposes, we decoded behavior (saccade vectors) from the motor output, based on physiological realism and previous network studies (Smith and Crawford 2005; Blohm et al. 2009). We chose to model saccades because of their simplicity and the availability of relevant data, but our model can be generalized to other visuomotor behaviors by adapting its input–output structure, for example to resemble the reach system (Blohm et al. 2009).

### Optimal allocentric–egocentric integration in behavior

In an early study that combined experimental data with statistical modeling, Byrne and Crawford (2010) showed that reach movements were biased toward shifted landmark when participants were instructed to reach to memorized visual targets in the presence of a visual landmark (presented as vibrating dots on the corners of an invisible square). The amount of shift was influenced by the actual relative reliability of gaze versus landmark positions, as well as the perceived stability of the visual landmarks: Higher stochasticity (induced by increasing the dots’ vibration range) resulted in less reliance on the landmark information. More recent experiments with more realistic scenes, replicated the basic result (i.e. aiming shifted with landmarks) but showed that the magnitude landmark influence was determined by different factors such as the number of shifted objects (Fiehler et al. 2014; Klinghammer et al. 2015) and scene consistency (Klinghammer et al. 2017). Similar effects have been observed in monkey gaze behavior (Li et al. 2017; Bharmauria et al. 2020).

Here, we showed that our network is capable of reproducing the observed monkey saccades with good agreement (*R*^2^ ≈ 75%–80%; Table 1, rows 1 and 2), as well as various other scenarios with different ego-allocentric weightings (Figs. 6 and 7). Our network reproduced similar allocentric weights as reported for monkey’s data (Fig. 6), but with wider distribution (Fig. 7). Some of the differences with real data might be accounted for by the existence of noise in sensorimotor systems (Körding and Wolpert 2006; Alikhanian et al. 2015; Abedi Khoozani and Blohm 2018). Consistent with this, increasing the noise reduced the ability of the network to explain data variability (Table 1, rows 3–6). This perhaps agrees with a recent report that the addition of noise to deep neural nets improves correspondence with actual response fields in early visual cortex (Jang et al. 2021).

How does the brain implement egocentric–allocentric integration? At a computational level, the predominant view in the field is that the brain uses probabilistic inference to estimate the reliability of ambiguous sensory signals for optimal integration (Ernst and Banks 2002; Alais and Burr 2004; Pitkow & Angelaki 2014). In this view, the brain estimates each signal contribution for the optimal integration (e.g. for allocentric–egocentric weighting) based on the statistical properties of sensory signals (where higher signal variability results in lower contribution). This can be derived online through probabilistic neural codes (Ma et al. 2006) or based on learned contextual cues (Mikula et al. 2018). For example, Byrne and Crawford (2010) explained their reach data using a maximum likelihood estimator based on the actual reliability of egocentric versus allocentric cues combined with a landmark stability heuristic based on prior experience. Here, our neural network model learned to extract such rules from the training set, consistent with the suggestions that error-based learning rules naturally implement probabilistic inference (Orhan and Ma 2017). But to understand how this is done, it is necessary to look within actual neural populations (Pitkow and Angelaki 2014).

### Simulation of neurophysiological results

As noted above, we coded the motor output of our network using the same motor population coding mechanisms as seen in the FEF (Bruce and Goldberg 1985; Sommer and Wurtz 2006; Sajad et al. 2015, 2016). This choice was made to (i) impose physiological realism within the model and (ii) allow direct comparisons of our network model with actual FEF data. To test if our motor output units generate similar properties as their counterpart motor neuron in FEF, we performed 3 analyses similar to neurophysiology studies. First, we confirmed that our motor population units show open-ended response fields and code information in a gaze-centered coordinate frame (Fig. 8). These observations are in line with previous observation of dominate gaze-centered coding in motor neurons (Sajad et al. 2015, 2016). Although, the open receptive field arose due to our design, the gaze-centered coding emerged from the training process. More specifically, although the overall population was expected to show geze-centered coding, individual units could have contributed to the output in many ways specially in the presence of multiple stimuli. For instance, we observed that in the presence of landmark, some units preferred landmark relative to the eye (Fig. 9, bottom row), suggesting that the landmark information made it all the way through the final output layer. This result suggests that observing Gfe as a dominate preferred coordinate frame is not trivial.

Second, and more importantly, when we examined the intermediate coordinate frames, we observed that our units use a range of intermediate coordinate frames between target and gaze to code the information as reported for FEF and SEF motor neurons. Third, examining the influence of the landmark shift, we observed that shifting the landmark resulted in shifted receptive fields (Fig. 9). In particular, we observed that the coordinate frames of our units were partially shifted toward the virtually shifted target (Fig. 10) representing an allocentric coding in our network. When we evaluated the allocentric–egocentric information, we found a strikingly similar partial integration as reported in SEF and FEF.

On the whole, these results suggest that we successfully created a model that can closely follow current neurophysiological reports (Bharmauria et al. 2020). This was a crucial goal for the current project. These observations suggest that the underlying mechanisms of the proposed network might have close similarities with brain mechanisms for allocentric–egocentric integration.

### Potential interpretability of the network processes

As suggested above, the striking similarities between our unit’s activity and their physiological counterparts suggest that our network might use similar mechanism as the brain to implement the required processes. This implies that interpretability of our network is essential. We argue that our network is highly interpretable by design. First, all the units of our CNN except, the feature pooling layer, are determined analytically; thus, they are fully interpretable. Moreover, the feature pooling also is mathematically fully measurable: A product of all the feature responses in the final maps determining crossing points of the 2 lines as well as the target location. The next and most challenging task will be to uncover the mechanisms governing the transformation *between* the input–output in our MLP. In particular, what happens in the trainable layers between our analytically defined visual system layers and motor output layer? For this purpose, it should be possible to use both analytic solutions arising from computer science (e.g. Hadji and Wildes 1990; Monga et al. 2020; Zhao and Wildes 2021) and “neurophysiological” techniques like those employed in the past (Zipser and Andersen 1988; Smith and Crawford 2005; Blohm et al. 2009). In this way, our visual-motor model can be used as a “quasi experimental model,” in parallel with studies on the real brain, for example to understand how egocentric and allocentric signals are integrated in goal-directed movements.

It is noteworthy that qualitative (i.e. visualization of convolutional filters) is significantly different from quantitative (i.e. mathematical specification) interpretability. Although visualizing the filters provides some insight into the working principles of CNNs, the learned convolutional filters, by definition, lack precise mathematical specification. Providing the mathematic specification of the convolutional filters, alongside the analytical specification of the rest of the hyperparameters, provides the possibility to mathematically calculate the processed image by the CNN and consequently to mathematically identify the CNN output. This is essential for the understanding of how the MLP implements the allocentric–egocentric integration (the focus of our next study).

### Limitations and future directions

Despite its success in replicating current neurophysiological and behavioral observations, this study is only the first step in modeling the integration of complex visual stimuli for sensorimotor transformations. To extend its usefulness beyond the current task (ego-allocentric cue conflict in the gaze system) several steps need to be taken. First, it should be possible to train this model on other 2D datasets (different visual stimulus configurations, etc.) to see how well it generalizes and investigate other questions involving the use of complex visual stimuli for movement control. Second, it should be possible to extend this model to include 3D geometry of sensorimotor transformation (and including other extra-retinal signals such as head position), multisensory (e.g. somatosensory) inputs of target and hand position. As emphasized by many studies, modeling the 3D linkage of eye–head–hand is crucial for a full understanding of sensorimotor transformations (for a review see Crawford et al. 2011). Third, another limitation of the current version of the model is that it does not include any temporal dynamics. A series of studies showed that FEF goes through a series of sensory–memory–motor transformations that include a dynamically evolving spatial memory signal (Sajad et al. 2015, 2016; Bharmauria et al. 2020, 2021). These studies classified recorded FEF neurons into 3 groups: visual neurons (neurons that are only active during the target onset), motor neurons (neurons that are active only during the gaze onset), and visuomotor neurons (neurons that are active during both target and gaze onset). Sajad et al. (2015, 2016) demonstrated that although visual neurons mostly code target in eye (the peak of the distribution is significantly shifted toward T_e_) and motor neurons code gaze in eye (the peak of the distribution is significantly shifted toward G_e_), visuomotor neurons deploy a distribution between T_e_–G_e_ with a peak being closer to T_e_ during the visual response and shifting toward G_e_ during the motor response. In our network, we only were able to analyze motor responses without having any visual or visuomotor responses. This resulted in a unimodal distribution in our T–G analysis (Fig. 11B (3)) as opposed to a bimodal distribution observed in monkeys’s FEF (Fig. 11B (6)). Therefore, temporal dynamics are essential for quantifying the progression of sensory coding through memory delay signals and into motor coding in such tasks. Including recurrent connection in the current model addresses this limitation. Finally, the current version of the model is appropriate for detecting simple stimuli such as 2D crosses or dots and therefore is not generalizable to datasets that include more realistic 3D objects. However, our studies in dynamic texture recognition show that incorporating additional CNN layers enables the current CNN model to recognize more complicated patterns (Hadji and Wildes 1990).

## Conclusions

We implemented and evaluated a neural network model—with physiologically constrained inputs and outputs—that provides both the capacity for encoding relatively complex visual features and sensorimotor transformation for goal-directed movements. We trained this model on real and synthetic datasets involving saccade generation in the presence of allocentric landmarks. We showed that our network replicates the reported behavior and generates the observed neural activities in FEF areas (i.e. dominance of gaze-center coding, emergent property of network design, as well as shifted coordinate frames and integration of allocentric–egocentric coordinate frames, emergent property of network training). These results suggest that our framework provides an analytic toolbox to better understand the interaction of allocentric and egocentric information for goal-directed movements. Specifically, having created, trained, and verified the model against real behavioral and neurophysiological data, further analysis of the hidden units in the MLP should be useful in understanding and predicting how the brain produces such transformations. We further propose that this network can be generalized to model other complex visuomotor tasks. Building such toolboxes is necessary to facilitate our further understanding of the underlying mechanisms for performing sensorimotor coordinate transformations where the stimulus is not simply a dot. Since spatial transformations ubiquitously underly most brain processes, this toolbox has potential for application in fields as diverse as reaching to grasp, posture/balance control, visual navigation, decision making, as well as their analogs in computer vision and robotics.

## Supplementary Material

Supplementary_Materials_tgac026
